# Effects of dietary carnitine supplementation on semen output and quality of boars

**DOI:** 10.1093/tas/txac143

**Published:** 2022-10-20

**Authors:** K B Balogun, N Lu, U Orlando, H Torborg, M Kleve-Feld, A Denton, A Holstine, K R Stewart

**Affiliations:** Department of Animal Sciences, Purdue University, West Lafayette, 47907 IN, USA; Genus PIC, Hendersonville, 37075 TN, USA; Genus PIC, Hendersonville, 37075 TN, USA; Kaesler Nutrition, Zeppelinstrasse 3, 27472 Cuxhaven, Germany; Genus PIC, Hendersonville, 37075 TN, USA; Department of Animal Sciences, Purdue University, West Lafayette, 47907 IN, USA; Department of Animal Sciences, Purdue University, West Lafayette, 47907 IN, USA; Department of Animal Sciences, Purdue University, West Lafayette, 47907 IN, USA

**Keywords:** boar, carnitine, sperm output, sperm quality

## Abstract

Carnitine is an amino acid derivative that performs the functions of increasing energy production as well as acting as an antioxidant for sperm cells. This study was conducted to investigate the effects of the inclusion of carnitine in boar diets on semen output and quality. Sixty-four purebred and hybrid boars at a commercial boar stud were blocked by age and semen quality and randomly allotted to receive a daily 30 g top-dress of either soybean meal (CON) or soybean meal and 625 mg of L-Carnitine (CARN). Supplementation lasted for 12 weeks from May to July 2021 during which weekly semen collection was performed. Semen was evaluated in the stud for concentration and motility parameters using computer-assisted semen analysis (CASA). Samples were shipped to Purdue University for detailed morphology, viability, and CASA analysis performed in samples stored at 17 °C for 5 days. PROC Mixed (SAS v 9.4) was used to analyze data, with boar nested within treatment used in repeated measures analysis. Semen quality estimates from the week before supplementation were used as covariates in the statistical model. Tukey–Kramer adjustment was used for means separation. Carnitine supplementation had no effects on total sperm produced (*P* = 0.35). Percentage of motile sperm cells (*P* = 0.63), morphologically normal sperm (*P* = 0.42), viable sperm (*P* = 0.43), or sperm with normal acrosomes (*P* = 0.61) in the ejaculates were not different among treatments. Sperm kinematics in CARN ejaculates tended to have greater straight-line velocity and distance (*P* = 0.06 and *P* = 0.07, respectively). There were several interactions of treatment and day of storage for the kinematic parameters. However, these interactions do not show observable trends for CARN to improve or depress sperm function. Overall, the inclusion of 625 mg/d of carnitine in the diet of boars for 12 weeks had no effects on sperm output or quality with minor changes to sperm cell kinematics.

## INTRODUCTION

The swine industry relies mainly on assisted reproductive technology, where greater than 90% of the industry uses artificial insemination for their sow breeding programs (Waberski et al., 2019). This involves the utilization of liquid stored semen from boars selected based on their genetic characteristics and ejaculate quality. As such, a large volume of high-quality sperm cells is expected of boars used for reproduction in swine production systems (Flowers, 2021). Factors that may negatively impact the quality of boar ejaculates include stress caused by season changes, management practices such as frequency of collection, and nutrition. Although meeting the energy and protein requirements of boars are important to produce good quantity semen and high-quality sperm cells, the addition of micronutrients to the boar diet has been reported to also be important (Wilson et al., 2004). Some of these micronutrients such as organic and inorganic minerals, vitamins, and amino acids, function as factors or co-factors in metabolic pathways important for spermatogenesis and sperm quality (Flowers, 2021).

Carnitine is an amino acid derivative and an essential metabolite that has a crucial role as a co-factor for the transportation of activated long-chain fatty acids from the cytosol to the mitochondria matrix, making it a necessity for energy metabolism in mammalian cells (Vaz and Wanders, 2002). This process is also believed to reduce the availability of lipids for peroxidation, thereby acting as a potent antioxidant (Neuman et al., 2002; Yang et al., 2020). Carnitine is mainly synthesized in the liver, and it functions to increase the production of adenosine triphosphate (ATP) within cells by facilitating the β-oxidation process (Agarwal and Said, 2004; Mongioi et al., 2016). The fact that carnitine is found in high concentration in the epididymis, the organ where sperm cells mature, gain motility, and utilize fatty acids and phospholipids as energy substrates, suggests that carnitine has an important role in the energy metabolism of sperm cells (Lenzi et al., 1992; Vicari and Calogero, 2001). Sperm cells need energy generated from their mitochondria for motility (Nesci et al., 2020). An increase in the supply of energy available to boar spermatozoa therefore may improve motility and the overall quality of boar semen. Carnitine has also been reported to trap excess mitochondrial acetyl-CoA as acetyl-L-carnitine, thereby protecting the activity of pyruvate dehydrogenase which has an important role in mitochondrial respiration (Mongioi et al., 2016).

In studies where L-carnitine has been added to cryopreserved human semen samples (Banihani et al., 2014) and liquid stored boar semen samples (Yang et al., 2020), motility has been reported to improve in both species and linked to the antioxidant properties and the role of carnitine in sperm ATP production. L-carnitine supplementation in diets of White Leghorn roosters (Neuman et al., 2002), Japanese quail breeders (Sarica et al., 2007), and Pietrain pigs (Yeste et al., 2010) have been reported to improve sperm concentration in the roosters, sperm viability in the Japanese quail, and sperm morphology in both roosters and the Pietrain pigs. Dietary carnitine supplementation may therefore improve boar sperm motility and overall boar semen quality by improving cellular energy metabolism and reducing boar sperm lipid peroxidation. The objective of this study was to investigate the effects of dietary supplementation of carnitine on semen output and quality of terminally crossbred boars. It was therefore hypothesized that the inclusion of carnitine in the diet of boars improves their semen output and quality.

## MATERIALS AND METHODS

The study was performed at a commercial boar stud in West Manchester, Ohio during the summer months of 2021. A total of 64 boars (PIC 337 and 800, initial age of 274 ± 21 days) were used in this trial. All experimental boars were individually housed in a crate, with a daily feed allowance of approximately 2.5 kg of a commercial corn and soybean meal-based diet (Table 1) and ad libitum access to water. Boars were blocked by genetic line and semen quality and randomly assigned to the control group (CON) or L-Carnitine supplemented group (CARN) at the start of the study. Boars in the CON were top dressed with 30 g of soybean meal per day; and boars in the CARN were top dressed with 30 g of soybean meal and the L-Carnitine product mixture to achieve approximately 625 mg of L-Carnitine intake per head per day. Top-dressing was imposed from May through July of 2021 (12 weeks), during which time semen was collected from boars according to the normal production schedule of the boar stud.

**Table 1.** Basal diet formulation and calculated composition

Ingredient	%
Corn	57.90
Wheat middlings	20.00
Soybean meal, 48%	12.00
Soybean hull	5.00
Dicalcium phosphate	1.47
Limestone	0.85
Corn oil	0.50
Salt	0.34
L-Lysine·HCl	0.25
L-Threonine	0.14
DL-Methionine hydroxy analog, 88%	0.13
Phytase	0.02
Vitamin and trace mineral premix^*^	0.66
Mycotoxin binder	0.45
Acidifier	0.30
Total	100
Calculated composition	
Metabolizable energy, kcal/kg	3116
Net energy, kcal/kg	2324
Crude protein, %	13.96
Ether extract, %	3.49
Standardized ileal digestible lysine, %	0.75
Standardized total tract digestible phosphorus, %	0.44
Analyzed calcium, %	0.90

^*^Supplied the following per kilogram of diets: 64.28 mg of manganese as manganese sulfate and manganese glycinate; 168.30 mg of iron as ferrous sulfate; 217.80 mg of zinc as zinc sulfate, zinc glycinate, and zinc hydrocloride; 32.25 mg of copper as copper sulfate, copper glycinate, and basic copper chloride; 0.45 mg of iodine as ethylenediamine dihydroiodide; 0.30 mg of selenium as selenium yeast and sodium selenite; 0.20 mg of chromium as chromium propionate; 28,660 IU of vitamin A; 4,409 IU of vitamin D_3_; 141 IU of vitamin E; 18 mg of vitamin K; 0.08 mg of vitamin B_12_; 15 mg of riboflavin; 66 mg of pantothenic acid; 309 mg of niacin; 4.4 mg of folic acid; 44 mg of vitamin B_6_; 8.8 mg of thiamin; and 0.41 mg of biotin.

Semen was routinely collected using the gloved-hand method from the study boars with an average of 6.1 days rest between collections. Immediately following collection (D0), semen was analyzed on the farm for concentration using a Libra S6 spectrophotometer (Biochrom, Holliston, MA) and motility using computer-assisted sperm assessment (CASA; SpermVision; MOFA Global, Verona, WI). Ejaculate volumes were measured using a digital gram scale. Total sperm production was calculated by multiplying semen volume by concentration. Semen samples from individual boars were then diluted in a commercial semen extender (NutrixCell+; IMV Technologies, Maple Grove, MN) to a final concentration of 37–40 million sperm cells/mL and packaged into semen tubes (Minitube, Venture Ct, Verona, WI). Semen tubes were cooled to 17 °C and one sample tube from each boar was transported to Purdue University (approx. 153 miles) by courier.

At Purdue, semen samples were maintained at 17 °C overnight. The next morning (Day 1), 1 mL of semen was warmed in a block heater at 37 °C for 20 min and evaluated by CASA (CEROS II, Hamilton Thorne, IMV Technologies, Maple Grove, MN) for motility and kinematic semen parameters including: Anterior Lateral Head Displacement (ALH); Beat-Cross Frequency (BCF); Average Path Distance (DAP); Curvilinear Distance (DCL); Straight-Line Distance (DSL); Linearity (LIN); Straightness (STR); Average Path Velocity (VAP); Curvilinear Velocity (VCL); Straight-Line Velocity (VSL); and Wobble (WOB). A separate 1 mL sample was preserved with 100 µL of 10% formalin and analyzed for morphological abnormalities and acrosome damage. Morphological abnormalities were assessed using phase-contrast, bright-field microscopy at 40× and classifying 200 cells as normal or containing distal cytoplasmic droplets, proximal cytoplasmic droplets, distal midpiece reflex, or head and tail abnormalities. Acrosome damage was assessed under oil immersion at 100× by classifying 100 acrosomal ridges as either normal or abnormal. Every fourth week of the trial, sperm cell membrane integrity was evaluated by propidium iodide staining using a Nucelocounter SP100 (Reproductive Provisions, Walworth, WI). To calculate the percentage of cells with intact plasma membranes, semen samples were evaluated twice on the Nucleocounter, once diluted in SP100 buffer to obtain total cell numbers and a second time diluted in semen extender to obtain the number of non-viable cells. The percent of viable cells was then calculated from these two numbers. Following evaluation on D1, semen was stored at 17 °C and re-evaluated on D3 and D5 for motility and sperm kinematic parameters using the same methods as described above.

All statistical analyses were performed using PROC MIXED of SAS v.9.4 with boar nested within treatment used in repeated measures analysis. For all variables, the main effects of treatment and week were included as fixed effects, with semen quality estimates from the week prior to supplementation included as covariates. All parameters measured were repeated by week with a compound symmetry covariance structure to minimize Akaike Information Criterion. A Tukey-Kramer adjustment was used as a means separation test. Statistical significance was defined as *P* ≤ 0.05 and a tendency at 0.05 < *P* ≤ 0.10.

## RESULTS

Semen data from prior to the feeding of carnitine is shown in Table 2. Results for the analysis of semen characteristics of boars fed with or without carnitine in daily diets are shown in Table 3. There were no significant differences in semen volume, viability, total sperm produced, agglutination, stud motility, and stud progressive motility (0.427 ≤ *P* ≥ 0.122) among treatment groups after 12 weeks of supplementing carnitine. There was however a tendency in concentration (*P =* 0.080). Concentration, volume, total sperm produced, stud motility, and stud progressive motility varied among the weeks of the feeding trial (*P* < 0.001), with no identifiable trend over time. Percent viable sperm cells (*P =* 0.645) and agglutination (*P =* 0.128) did not vary by week of analysis. Treatment by week interactions was not significant for most variables (0.870 ≤ *P* ≥ 0.176), except for a tendency in semen volume (*P* = 0.080).

**Table 2.** Semen data before carnitine feeding started

Variables	Treatment		*P*-value
	CON	CARN	
Age, days	250.12 ± 3.17	249.45 ± 3.10	0.881
Quality, % abnormal cells	8.67 ± 0.39	9.00 ± 0.78	0.702
Sperm Output, ×10^9^	52.66 ± 2.59	56.11 ± 3.35	0.418

CON—control animals, fed 30 g top-dress of corn daily.

CARN—treatment animals, fed corn and 625 mg of carnitine daily.

**Table 3.** Semen sample characteristic variables least-square means ± SE of boars fed daily diets with or without 625 mg of carnitine

Variables	Treatment		*P*-value		
	CON	CARN	Treatment	Week	Treatment × Week
Concentration, ×10^6^/mL	60.08 ± 2.73	67.42 ± 3.77	0.080	<0.001	0.283
Volume, mL	155.45 ± 2.55	143.95 ± 2.26	0.122	<0.001	0.080
Viability, %	84.86 ± 0.47	83.92 ± 0.58	0.427	0.645	0.176
Total sperm produced, ×10^9^	74.47 ± 1.29	78.96 ± 1.53	0.349	<0.001	0.870
Agglutination, 1–3	1.72 ± 0.13	1.56 ± 0.04	0.324	0.128	0.853
Stud motility, %	84.08 ± 0.40	84.99 ± 0.35	0.377	<0.001	0.565
Stud progressive motility, %	74.50 ± 0.56	76.31 ± 0.45	0.167	<0.001	0.457

CON—control animals, fed 30 g top-dress of corn daily.

CARN—treatment animals, fed corn and 625 mg of carnitine daily.

The results of the CASA analysis are shown in Table 4. No significant differences were found between CON and CARN boars in motility, progressive motility, or any sperm kinematic measures (0.976 ≤ *P* ≥ 0.101), except for tendencies for some kinematic measures like DSL (*P* = 0.066), STR (*P* = 0.093), and VSL (*P* = 0.055). Day of storage impacted motility and most kinematic parameters (*P* < 0.001), except for a tendency in percent wobble (*P* = 0.096). Motility, progressive motility, and all kinematic measures varied by the week of the study (*P* < 0.001), with no observable trends. Treatment by week interaction effects was not significant for motility (*P* = 0.796) but had a tendency in progressive motility (*P* = 0.086). All kinematic measures were significant for treatment by week interactions (0.021 ≤ *P* ≥ 0.001, Table 4). Treatment by day interaction effects were significant only for BCF (*P =* 0.048), DAP (*P* = 0.038), and DSL (*P* = 0.033). All treatment by week by day effects were significant (*P* < 0.001).

**Table 4.** Sperm motility and kinematic variables least-square mean ± SE of liquid stored semen samples of boars fed with or without 625 mg of carnitine in daily diets

Variables	Treatment			Day		
	CON	CARN		Day 1	Day 3	Day 5
Motility, %	82.63 ± 0.25	81.99 ± 0.24		80.53 ± 0.34^c^	83.88 ± 0.26^a^	82.51 ± 0.30^b^
Prog. Motility, %	62.67 ± 0.36	59.61 ± 0.36		58.82 ± 0.48^b^	62.19 ± 0.42^a^	62.41 ± 0.42^a^
ALH, μm	6.02 ± 0.03	6.03 ± 0.03		6.17 ± 0.03^a^	6.04 ± 0.03^b^	5.87 ± 0.03^c^
BCF, Hz	34.47 ± 0.07	34.40 ± 0.07		34.00 ± 0.08^b^	34.65 ± 0.08^a^	34.67 ± 0.08^a^
DAP, μm	40.03 ± 0.14	39.58 ± 0.15		39.27 ± 0.18^b^	40.24 ± 0.17^a^	39.91 ± 0.17^a^
DCL, μm	70.52 ± 0.30	71.18 ± 0.34		70.05 ± 0.41^b^	71.79 ± 0.40^a^	70.71 ± 0.36^b^
DSL, μm	28.38 ± 0.15	27.00 ± 0.15		26.89 ± 0.18^b^	27.86 ± 0.18^a^	28.33 ± 0.19^a^
LIN, %	44.92 ± 0.25	43.09 ± 0.26		43.34 ± 0.33^b^	43.82 ± 0.32^b^	44.86 ± 0.30^a^
STR, %	73.63 ± 0.23	71.65 ± 0.24		71.65 ± 0.30^c^	72.54 ± 0.30^b^	73.73 ± 0.28^a^
VAP, μm/s	77.18 ± 0.29	75.72 ± 0.28		75.66 ± 0.38^b^	77.37 ± 0.33^a^	76.31 ± 0.34^b^
VCL, μm/s	134.34 ± 0.61	134.30 ± 0.62		133.15 ± 0.79^b^	136.29 ± 0.76^a^	133.51 ± 0.69^b^
VSL, μm/s	57.12 ± 0.27	54.36 ± 0.26		54.44 ± 0.34^b^	56.25 ± 0.31^a^	56.50 ± 0.32^a^
WOB, %	59.08 ± 0.17	58.10 ± 0.18		58.48 ± 0.23^b^	58.43 ± 0.21^c^	58.85 ± 0.20^a^
Variables	*P*-value					
	Treatment	Day	Week	Treatment × week	Treatment × day	Treatment × week × day
Motility, %	0.625	<0.001	<0.001	0.796	0.425	<0.001
Prog. Motility, %	0.101	<0.001	<0.001	0.086	0.629	<0.001
ALH, μm	0.877	<0.001	<0.001	<0.001	0.794	<0.001
BCF, Hz	0.805	<0.001	<0.001	0.001	0.048	<0.001
DAP, μm	0.404	<0.001	<0.001	0.001	0.038	<0.001
DCL, μm	0.580	<0.001	<0.001	<0.001	0.109	<0.001
DSL, μm	0.066	<0.001	<0.001	0.002	0.033	<0.001
LIN, %	0.166	<0.001	<0.001	<0.001	0.453	<0.001
STR, %	0.093	<0.001	<0.001	0.001	0.741	<0.001
VAP, μm/s	0.216	<0.001	<0.001	0.021	0.728	<0.001
VCL, μm/s	0.976	<0.001	<0.001	0.004	0.931	<0.001
VSL, μm/s	0.055	<0.001	<0.001	0.003	0.416	<0.001
WOB, %	0.274	0.096	<0.001	<0.001	0.301	<0.001

a^,^b^,^cAre used to indicate significant differences among means within rows.

CON—control animals, fed 30 g top-dress of corn daily.

CARN—treatment animals, fed corn and 625 mg of carnitine daily.

Prog. motility—progressive motility.

Anterior Lateral Head Displacement (ALH); Beat-Cross Frequency (BCF); Average Path Distance (DAP); Curvilinear Distance (DCL); Straight-Line Distance (DSL); Linearity (LIN); Straightness (STR); Average Path Velocity (VAP); Curvilinear Velocity (VCL); Straight-Line Velocity (VSL); Wobble (WOB).

Sperm morphology analysis results are shown in Table 5. Sperm cell and acrosome morphology did not differ among treatments (0.832 ≤ *P* ≥ 0.219). Variation in normal morphology was seen among the weeks of the study but with no observable trend. A tendency was recorded in intact acrosomes (*P =* 0.057). However, no treatment by week interactions were present in most variables (0.205 ≤ *P* ≥ 0.744), suggesting that most abnormalities in sperm cells varied equally across treatments. There were nevertheless tendencies in normal cells (*P =* 0.062), and head and tail abnormalities (*P* = 0.070).

**Table 5.** Sperm morphology variables least-square means ± SE of liquid stored semen samples of boars fed with or without 625 mg of carnitine in daily diets.

Variables, %	Treatment		*P*-value		
	CON	CARN	Treatment	Week	Treatment × week
Normal cells	90.98 ± 0.43	89.97 ± 0.36	0.422	<0.001	0.062
Proximal droplets	2.36 ± 0.17	2.50 ± 0.20	0.832	0.008	0.245
Distal droplets	3.10 ± 0.16	3.84 ± 0.20	0.219	<0.001	0.205
Distal midpiece reflex (DMR)	1.55 ± 0.12	2.06 ± 0.23	0.380	0.380	0.744
Head and tail abnormalities	2.05 ± 0.31	1.64 ± 0.07	0.322	<0.001	0.070
Intact acrosomes	95.62 ± 0.41	95.39 ± 0.15	0.613	0.057	0.713

CON—control animals, fed 30 g top-dress of corn daily.

CARN—treatment animals, fed corn and 625 mg of carnitine daily.

## DISCUSSION

The objective of this study was to evaluate the effects of utilizing dietary carnitine for the improvement of output and quality of boar semen. Carnitine is available in high concentration in the epididymis because of the important role it has in the metabolism and maturation of sperm (Agarwal and Said, 2004). It facilitates the transport of long chain fatty acids through the inner membrane of the mitochondria for metabolic processes that generate intracellular energy. Also, high concentrations of carnitine in testicles and acetyl-L-carnitine transferase in primary spermatocytes and testicular tissue in rats have been reported (Schanbacher et al., 1974). Its effect on germ cell maturation is evident in the increased secretion of essential energy substrates (pyruvate and lactate) when L-carnitine was added to Sertoli cell cultures (Palmero et al., 2000). The tendency for dietary carnitine to improve the concentration of boar spermatozoa in the present study may be because of the increase in energy substrates needed during spermatogenesis to produce spermatids and spermatozoa. This result agrees with Neuman et al. (2002) who reported an increase in sperm concentration of white leghorn roosters after 3–4 weeks of feeding dietary carnitine at 500 mg/kg. This research group associated the antioxidative properties of carnitine with the protection of sperm lipid membranes. The antioxidant property of carnitine has been reported to be exerted through a repairing mechanism that lowers the level of toxic intracellular acetyl-CoA, coupled with the replacement of membrane phospholipids fatty acids in some cases (Vicari and Calogero, 2001). Zhai et al. (2007) also reported a similar and consistent increase in sperm concentration when a lower inclusion level of carnitine, 125 mg/kg of feed, was fed to roosters from hatch until 37 weeks.

There was no significant difference observed in the total sperm produced by boars fed 625 mg of carnitine per day. However, Kozink et al. (2004) reported that feeding adult, but not young, post-pubertal boars with 500 mg L-carnitine per day increased total sperm produced. Sperm cell viability as measured by membrane integrity was not impacted by dietary carnitine, which explains the lack of differences in agglutination of semen samples as one of the leading causes of agglutination is from sperm cells with damaged plasma membranes that become “sticky” causing additional sperm cells to clump together. The supplementation of boar diet with 625 mg of carnitine for 12 weeks in this study was expected to be sufficient to exert its effect, if there is any, since the average duration of the spermatogenesis process in boars is approximately 6 weeks (França et al., 2005). 

Motility and progressive motility were not impacted after feeding boars 625 mg of carnitine daily for 12 weeks. We hypothesized that since carnitine has a crucial role in generating cellular energy in the mitochondria matrix of sperm cells, and this energy is important for motility, there would be an improvement in sperm motility of carnitine fed boars. Our finding is however in agreement with Jacyno et al. (2007) who fed 500 mg of L-carnitine daily to Pietrain boars for 5 weeks and reported no improvement in motility. Ahangari et al. (2012) however reported improvement in the motility of Japanese quail sperm cells with dietary supplementation of L-carnitine at 125 mg/kg for 35 days and treating asthenozoospermic men with 2 g oral L-carnitine daily significantly increased percentage sperm motility after 3 months (Garolla et al., 2005). Stradaioli et al. (2004) also reported a similar increase in progressive motility in stallions fed L-carnitine. However, their findings revealed that normospermic stallions show no observable improvement in semen quality compared with stallions with low sperm motility and other semen characteristics when fed L-carnitine at 20 g for 60 days. Perhaps, the administration of carnitine would have been more effective in restoring semen quality if fed to boars with already established compromised semen quality. Flowers (2021) posited that supplementation of diet with micronutrients may be more effective in reversing the negative impact of factors such as frequent semen collections, heat stress, and other similar kinds of stressors, than improving the existing quantity and quality semen produced. This claim is supported by the findings of studies where betaine and arginine supplementation proved to improve semen quantity and quality of heat-stressed boars (Cabezón et al., 2016; Chen et al., 2018). Conversely, the ineffectiveness of oral administration of carnitine on boar sperm motility and progressive motility in the present study may also be due to the dosage of carnitine used. Perhaps, a higher concentration of carnitine may have resulted in observable improvement in spermatozoa motility and progressive motility in boars. The fact that motility and progressive motility were higher on days 3 and 5 than on day 1 shows the impact that transportation-induced stress may have had on the sperm cells of boar semen doses seen on D1. Similarly, sperm kinematic parameters including BCF, DAP, DSL, VSL, and STR were higher on D3 and D5 than on D1. This agrees with the finding of an earlier study where motility and progressive motility of extended boar semen were higher on day 3 of storage than in day 1 (Balogun and Stewart, 2021).

## CONCLUSION

The effects of dietary carnitine on boar semen output and quality were investigated in this study. The major finding of this study however is that the supplementation of 625 mg L-Carnitine in the daily ration of boars for 12 weeks did not impact sperm output or quality but had minor effects on sperm cell kinematics.
